# Rare disease registries: potential applications towards impact on development of new drug treatments

**DOI:** 10.1186/s13023-018-0836-0

**Published:** 2018-09-05

**Authors:** Marijke C. Jansen-van der Weide, Charlotte M. W. Gaasterland, Kit C. B. Roes, Caridad Pontes, Roser Vives, Arantxa Sancho, Stavros Nikolakopoulos, Eric Vermeulen, Johanna H. van der Lee

**Affiliations:** 1 Pediatric Clinical Research Office, Emma Children’s Hospital, Academic Medical Center, University of Amsterdam, Amsterdam, The Netherlands; 2 Department of Biostatistics and Research Support, Julius Center for Health Sciences and Primary Care, University Medical Center Utrecht, University of Utrecht, Utrecht, The Netherlands; 3 Clinical Pharmacology Unit, Parc Taulí Hospital Universitari. Institut d’Investigació i Innovació Parc Taulí I3PT, Sabadell, Spain; 4 Servicio de Farmacología Clínica – Hospital Universitario Puerta de Hierro Majadahonda, Madrid, Spain; 5 Academic Medical Center, H8-236, Meibergdreef 9, 1105 AZ Amsterdam, The Netherlands

**Keywords:** Rare disease, Registry, Clinical trial, Drug development

## Abstract

**Background:**

Low prevalence, lack of knowledge about the disease course, and phenotype heterogeneity hamper the development of drugs for rare diseases. Rare disease registries (RDRs) can be helpful by playing a role in understanding the course of the disease, and providing information necessary for clinical trial design, if designed and maintained properly. We describe the potential applications of a RDR and what type of information should be incorporated to support the design of clinical trials in the process of drug development, based on a broad inventory of registry experience. We evaluated two existing RDRs in more detail to check the completeness of these RDRs for trial design.

**Results:**

Before and during the application for regulatory approval a RDR can improve the efficiency and quality in clinical trial design by informing the sample size calculation and expected disease course. In exceptional circumstances information from RDRs has been used as historical controls for a one-armed clinical trial, and high quality RDRs may be used for registry-based randomized controlled trials. In the post marketing phase of (conditional) drug approval a disease-specific RDR is likely to provide more relevant information than a product-specific registry.

**Conclusions:**

A RDR can be very helpful to improve the efficiency and quality of clinical trial design in several ways. To enable the applicability and optimal use of a RDR longitudinal data collection is indispensable, and specific data collection, prepared for repeated measurement, is needed. The developed checklist can help to define the appropriate variables to include. Attention should be paid to the inclusion of patient-relevant outcome measures in the RDR from the start. More research and experience is needed on the possibilities and limitations of combining RDR information with clinical trial data to maximize the availability of relevant evidence for regulatory decisions in rare diseases.

## Background

In rare diseases the clinical development of effective drugs is challenging due to low prevalence of the disease and often considerable phenotype heterogeneity. The small numbers give limited opportunity for confirmatory clinical trials, as it is difficult to recruit sufficient patients [1]. For many rare diseases the disease course is insufficiently known, leading to uncertainties with regard to the optimal clinical trial design, including choice of endpoints, for a new drug. Even if the endpoints are clear, there is often insufficient information about their occurrence - in case of binary endpoints-, or distribution - in case the endpoint is a continuous variable. This lack of information, combined with heterogeneity, has consequences for the efficiency of preparing and designing a clinical trial. More specifically, assessing the feasibility of a trial, which is directly connected with robust sample size calculations, becomes a difficult task. When the sample size is calculated based on limited information, this increases the risk of under- or overestimation of the sample size, and possibly a failed trial [2, 3]. Furthermore, such scarcity of information can have considerable impact on the regulatory process and evaluation of the available evidence for the risk/benefit assessment of a new drug, and possible market authorization [4].

In this respect rare disease registries (RDRs) can be an invaluable source of information. With an estimated number of 5000–7000 distinct rare diseases, in Europe over 700 RDRs are active for a similar number of rare diseases [5, 6]. A RDR can give insight in the natural history of the disease and the variability of the patient population. The decision to start a RDR is often made at a stage when the available information about the particular rare disease is still scarce, and the development of a treatment may lie far away in the future. Even in such an early phase, it is important to be aware of and prepared for all possible future functions of the information contained in the RDR; the identification of relevant endpoints or the use as a data source for the design of a therapeutic clinical trial are two notable examples [7]. Therefore, a RDR should be designed in such a way that all relevant information is incorporated and can be used most efficiently. The term ‘registry’ can denote any type of data collection. However, not all data collections that are presented as disease registries are suitable for clinical trial design or as additional information for regulatory evaluation. Several types of registries can be distinguished, and there is no consensus on the nomenclature and classification.

In a study for the EPIRARE project, addressing regulatory, ethical, technical and financial issues related to the development of RDR, registries were classified into three clusters: public health registries, clinical and genetic registries, and treatment registries [8]. Public health registries are aimed at epidemiological research, healthcare service planning, and disease surveillance. These registries generally are population based and collect information on more than one disease or condition, for example on cancer or congenital anomalies [9, 10]. Clinical and genetic registries focus on etiological research questions. They collect information on phenotype, genotype, family history and clinical data. Treatment registries are predominantly aimed at treatment evaluation and monitoring. For example, in registries for post-marketing surveillance, often required by regulators for (conditional) market approval, information is collected on outcomes from patients who use a particular medicinal product. Often, these registries are focused on one, or few, specific treatments. Not all types of registries collect the appropriate information to be useful in clinical trial design or drug development. For instance, the information collected in population-based registries often is not specific enough to inform natural course and relevant disease-specific outcomes. The same holds for genetic and clinical registries in which no outcomes are collected at a regular basis. In the User’s Guide of the US Agency for Healthcare Research and Quality, aimed at registries that evaluate patient outcomes, but not limited to rare disease registries, a different categorization is used [11]. Registries are divided into product or health service registries, patient or disease registries, and combinations of these. Product registries generally focus on the determination of (cost-) effectiveness, quality of care, and safety and harm of a product and only contain information on individuals who make use of a particular product or set of products. Patient or disease registries focus on natural history, but could also be used for collecting information about efficacy and/or safety of interventions [11]. Here, we define RDRs as standardized data collections including information about patients with a particular rare disease, without selection based on treatment received.

The EPIRARE project defined a set of common data elements (CDEs) to improve standardization and data comparability among RDRs and to support new registries and data collections. This set of CDEs is intended to provide the basic elements for the construction of registries for a variety of purposes, but their main focus is on population-based RDRs, such as public health registries. Besides a mandatory set of baseline elements, such as demographic characteristics and recruitment information, other domains can be chosen accordingly to the RDR’s purpose [12]. When setting up a RDR for a certain rare disease with the intention to use the information for clinical research later on, additional, disease-specific data elements, not included in the CDEs, may be necessary. For example, in chronic, slowly deteriorating rare diseases mortality and quality of life (advised as CDEs) might not be specific enough when describing the disease course and testing the efficacy of a drug at a later stage.

In this paper, we describe possibilities for the applications of disease-specific RDRs for clinical trial design and drug development in rare diseases, and we provide suggestions about which data elements at least should be collected for that purpose. We also give recommendations for the optimal design of RDRs to enhance interventional research, including trials for regulatory approval, in the future.

## Methods

This overview was developed by experts from the Asterix consortium [13]. Asterix is a EU funded consortium that focuses on the development of research methodologies for rare disease drug trials. The Asterix team comprises statisticians, methodologists, patient representatives, regulators, and clinicians, all with expertise in the field of rare diseases. We first conducted focus groups and interviews to investigate the RDR applications and to develop a checklist of elements to record. Possible relevant information from models from the literature was added to the checklist, which was discussed by e-mail until there was consensus. Finally, we checked the completeness of two existing RDRs for trial design.

### Focus groups, interview, European Public Assessment Reports

First, we conducted two focus groups with 3 different statisticians each and an interview with two regulatory experts with extensive experience at the European Medicines agency (EMA), all involved in the Asterix project. The principal question was in what way a RDR could be informative for the design of a pivotal clinical trial for regulatory approval and what information should be included to serve this goal. Minutes were taken from both focus groups and circulated among the participants for feedback. The interview with the regulatory experts was recorded and the summary report was also checked by the participants for completeness and correctness. The overview of possible applications of a RDR in trial design and the checklist of elements to be recorded, was based on the reports of the focus groups and the interview. The overview was completed with selected European Public Assessment Reports (EPARs) describing examples in which RDR data had been used for drug approval.

### Draft checklist and consensus

A first draft checklist of data elements to incorporate in a disease-specific RDR for trial purposes was made. In a literature review, two models were found describing general domains of data elements in rare disease registries and/or outcome measures which we compared with our draft checklist. One model gives recommendations for general data elements for rare disease registries (EPIRARE) [12], the other model describes domains of outcome measures as a basis for the choice of core outcome sets (OMERACT) [14]. Both models did not provide a complete overview on variable domains to include in a disease-specific RDR for enhancement in trial design and drug approval. Therefore, we merged possible relevant information from these two models with our draft checklist. The checklist was completed with information on the frequency of and reasons for data collection, and the applicability in the drug approval process. This version of the draft list was circulated among the consulted experts in order to obtain consensus on the final checklist.

### Evaluation of existing RDRs

The final checklist was used to evaluate whether all necessary elements to inform a clinical trial in the future were included in two existing disease-specific RDRs. The two available disease-specific RDRs selected were the European Cystic Fibrosis Society (ECFS) registry and the Diffuse Intrinsic Pontine Glioma (DIPG) registry. These two RDRs were selected from eight RDRs whose coordinators had participated in interviews about the goals and set-up of RDRs, and their use in clinical trials. The selection was based on variability in disease, their possible use of RDR data in clinical research or trials, and the possibility to retrieve the data elements collected in the RDR. Possible gaps or differences between the RDR and the checklist were highlighted and suggestions for improvement were given.

## Results

The importance of RDR information for the clinical development stage of a medicinal product for rare diseases was endorsed by all experts. The possible use of a RDR was divided in five main categories, i.e. 1) general aspects of a RDR for research of the particular disease, 2) the possible application of a RDR for sample size calculations and sample size reduction, 3) the use of a RDR for a registry-based clinical trial, 4) the use of RDR information as a historical control group, and 5) possible application of a RDR in the post-marketing phase (safety, off-label use, continued assurance of effectiveness). Also, an elaboration was given on the requirements of a RDR to be applicable in trial design, followed by the results from the developed checklist.

### 1) General aspects of a RDR

A RDR can give insight in the natural course of the disease, providing good starting points for relevant research. Registries are particularly relevant for rare diseases, as they may often be the main source of knowledge. In the phase of protocol development and regulatory scientific advice, the knowledge gathered from the RDR forms the foundation for many relevant features of the development plan (and the clinical trial design), such as information on prevalence, clinical course of disease, prognostic subgroups and relevance of surrogate endpoints (when collected) or other outcome measures [7]. Through the RDR, sites with expertise in managing the disease and patients who may be eligible for a trial can be located, which may allow to estimate the trial feasibility and can enhance the efficiency once the trial is open for recruitment.

### 2) Possible application of a RDR for sample size calculations and sample size reduction

For the design of future clinical studies, RDRs may provide a database of prior information that can be used in different ways. The most direct use of this information would be as input for estimations of nuisance parameters to inform sample size calculations for a new trial. Nuisance parameters reflect aspects of the probability distributions that affect the precision of estimators of the target parameter, but are not of primary interest. Examples of nuisance parameters are the standard deviation (for continuous outcomes) and the control event rate (for dichotomous outcomes). Empirical information on the nuisance parameters gives reliable input for sample size calculations [15]. The availability of this information alleviates the need for pilot studies, thus saving time and resources. Sometimes the registry data (or part of the data) might be used directly as part of the (Bayesian) design and analysis of a new study, which may reduce the necessary sample size. This can be done in several ways – add data (e.g. on the control treatment) as “pseudo observations” in the new trial, combine part of the RDR data and data from the new trial in a Bayesian analysis or use the registry to model disease progression and use this as reference in the trial [7]. This is not without controversy among trial methodologists and regulators. However, in (very) rare diseases this approach might be preferable to conducting a trial that is essentially too small, and then synthesize the evidence of this trial in a less formal way, e.g. by post-hoc analyses, after the trial is completed.

### 3) The use of a RDR for a registry-based clinical trial

Registry-based randomized controlled trials (RCT) use registries as a platform for case records, data collection, randomization, and follow-up. From the registry database the eligible patients are randomly assigned to receive the intervention or control treatment, and both the baseline and follow up data already collected in the registry form the basis for the RCT [16]. These trials have many advantages over traditional RCTs, such as lower use of resources, higher rate of enrolment of patients, and, besides potential completeness of the baseline data, enhanced generalizability of findings, as with this structure assessment of patients who are or are not participating in the trial is possible. The advantages of this approach in very to ultra-rare diseases are debatable. In case of ultra-rare diseases or within-disease rarity through selection of specific genotypes, randomization might not be viable. Also, although a registry-based RCT saves money, keeping up a high-quality RDR is expensive, so it is questionable whether in the end this approach is financially favourable for rare diseases. On the other hand, for financial and logistic reasons pharmaceutical companies may be more willing to start a RCT when this could be embedded in the already existing RDR.

Registry-based randomized controlled trials may face some ethical issues. One of these is the design of the consent procedure. Is a formal informed consent for the ‘control’ treatment needed when a patient has consented the data collected in the registry could be used comparatively? In a study by Relton et al., among a cohort of women with menopausal hot flushes a Zelen design for the consent procedure was used. At the time of random allocation of patients to the active and control arm, only the patients assigned to the active treatment were informed about this [17]. After this trial the opinion of the ‘control’ patients on this procedure had not been evaluated. Although some patients might just be fine with not knowing, others may feel not-informed and left out of the loop. However, both the Declaration of Helsinki and the ICH Guidance on Good Clinical Practice are clear in that the subject must receive detailed explanations on the experimental nature and design of the trial, including its purpose, the tested treatments and the probability for random assignment to each treatment [18, 19]. Also another study mentions the importance of informed consent in pragmatic randomization [20].Whether a general acceptance of data collection in a RDR for comparative use may overcome such recommendations can be controversial. So, these (among other) challenges have to be taken into consideration before proper execution can take place [21].

### 4) The use of RDR information as a historical control group

The RCT with an appropriate comparator arm is the design of first choice, as is also stated in the EMA guideline on clinical trials in small populations [22]. The random allocation minimises selection bias and the concurrent comparison group allows the researchers to determine any effects of the treatment compared with the control treatment unbiased by the effects of unmeasured confounders [23]. With regard to rare diseases, randomized studies can still be conducted, as in many diseases the patient population is large enough to perform successful random allocation. However, in certain circumstances, depending on type of disease and treatment possibilities, rare diseases may introduce complexity in the consideration whether a RCT is a viable option. For instance when a disease is ultra-rare and severely progressive for which no treatment is available yet, ethically sound alternatives might be considered to open the door for drug development. One of these alternatives is the possibility of using the data of RDR patients as historical controls for a single arm trial. Below, two examples are described of drugs approved after historical data had been used as a control group for a single arm pivotal study.

#### Example 1

In 2006 the new drug Myozyme was approved by the EMA for Pompe’s disease, a rare autosomal recessive lysosomal storage disease. This decision was based on data from studies among patients with late-onset and infantile-onset Pompe’s disease. These studies included, among others, a double-blind, randomized, placebo controlled study with a duration of 18 months, involving 90 patients with late-onset Pompe’s disease [24, 25], and a single-arm trial among 9 children with infantile-onset Pompe’s disease [26, 27]. Because this infantile-onset disease is a rapidly fatal disorder and former information already showed a beneficial effect of the drug, the use of a placebo arm was considered unethical. Instead, an untreated cohort of infantile-onset Pompe’s disease patients from the disease registry, fulfilling the inclusion criteria of the trials, was identified to serve as a reference group. Based on these results Myozyme was authorized both in late-onset and infantile onset Pompe’s disease [28–30].

#### Example 2

The drug Defitelio is used to treat severe veno-occlusive disease (VOD) in patients undergoing hematopoietic stem-cell transplantation. VOD is a condition in which the hepatic veins become blocked, leading to liver dysfunction. VOD is known to have a high mortality rate (between 75 and 85%). In one main study including 102 patients with VOD, the mortality rate after treatment with Defitelio was compared to a historical control group of patients who had received standard supportive care. The mortality rate in the group treated with Defitelio was 62% compared to 75% in the historical control group. The committee for medicinal products for human use (CHMP) concluded that Defitelio’s benefits were greater than its risks [31]. So, despite lack of a concurrent placebo comparison, the results were convincing enough for market authorization, but under ‘exceptional circumstances’. This meant that, because it was not possible to obtain complete information, a registry including the patients receiving the treatment was required to provide further data on safety, health outcomes, and the way the drug is used in practice.

### 5) Possible application of a RDR in the post-marketing phase

Applying RDR data in the post-marketing phase is useful for the systematic collection of real-world data on the use of new treatments. The participants in a trial usually constitute a selection of relatively homogeneous and fit patients (although in rare diseases this is less the case), who are selected based on genotype or other disease aspects and are closely followed and controlled. When based on the trial it is concluded that the intervention has shown benefit in such a patient population, it is under the relatively artificial conditions of the trial, and generally for a shorter period of time than the intended time that patients will be treated in clinical practice. Thus, at the time of marketing approval, the information on safety and effectiveness of the product is inferred to be applicable also to many other patients with different characteristics and for long term treatments. With regard to rare diseases, valid information from a RDR may provide evidence to clear uncertainties on the effectiveness and safety of the product in conditions of routine clinical practice and in wider patient populations. This is illustrated by an example of Elaprase, a drug used as enzyme-replacement therapy in Hunter syndrome. Based on a placebo-controlled trial among 96 patients aged 6 to 31 years old, market authorization was given. However, as of limited data, follow-up information was requested to investigate the long-term effects of the drug [32]. Because the clinical trial was restricted to patients from 6 years onwards, no efficacy information was available for younger patients. A broad age range of patients enrolled in the registry allowed an analysis to be performed of safety and preliminary clinical outcomes in patients younger than 6 years of age. Findings from the registry, together with an open-label study among young patients, supported marketing authorization of Elaprase in children from this younger age group [33, 34]. Also in Gaucher’s disease long-term treatment information was obtained on enzyme-replacement therapy through the International Collaborative Gaucher Group Gaucher Registry [35, 36]. This registry has been useful to confirm benefits on clinical outcomes and long-term effectiveness of the treatment in routine clinical practice, including wider patient populations than those included in clinical trials.

### Requirements of a RDR to be useful for trial design

In the design phase of a RDR it is important to consider the research questions and endpoints of a possible clinical trial in the future. By considering a possible future trial it becomes clear what information one would preferably be able to extract from the registry in the future. Information on the primary outcome is necessary to calculate the necessary trial sample size. This means that it is important to collect the right type of information on potential primary outcomes in the right way from the beginning of the registry. One needs to think about the use of validated measurement instruments, or if necessary further validation of promising instruments, and the timing and performance of the measurements must be standardized, preferably internationally. This is needed to provide a reliable source of information and to increase the chance of successful use for a clinical trial.

To leverage use of prior information through Bayesian analysis, the preferred path would be that prior distributions for the (efficacy) parameters of interest – such as change in disease severity or event incidences – can be based on actual data, rather than prior beliefs. A RDR may provide such data, but then needs to register the outcomes that will be of interest in the clinical trial to be designed, as well as include broad variability in the patient population registered to be able to represent all possible patient groups in a future trial. Preferably patients are included in all disease progression states, from multiple centers and internationally, with standardized outcomes. This is necessary for the, essential, exchangeability assumption to be fulfilled, which means that the prior information is based on a population that is comparable to the one included in the trial.

For the use of RDR data in a registry-based clinical trial, as a historical control group for clinical trials or in the post-marketing phase, similar considerations are important as for its use in a sample size calculation. With regard to the registry-based RCT a high-quality database is a prerequisite including flexibility to add variables if necessary, as well as an adequate number of patients who are eligible for the registry- based RCT. There are three main concerns with the use of historical data. The first is the comparability of the trial participants with the patients in the RDR, and the second is the comparability of the collected data. The third is the evolving standard of care that may induce bias when the control group is not concurrent. To overcome the first two issues, standardization of measurements and data collection from the very start of the registry is paramount, and measures intended to check comparability and allow matching by baseline variables that may be related with patient evolution over time, such as severity of the disease, age, and genotype, need to be available. Again, sufficient patients need to be included in the registry to enable a useful selection of patients allocated as comparator group.

For all applications of RDR information mentioned above, longitudinal data collection, preferably in prospectively defined intervals, is key. This means that information on the outcome measure needs to contain at least two points in time. In this way the development of the outcome measure can be assessed for all patients, irrespective of what treatment they received. In its most simple form, this could be date of birth and date of death in order to assess mortality in a severe lethal disease, such as DIPG. A more complex example is the 1 min forced expiratory volume (FEV1) in patients with cystic fibrosis, which is regularly measured in all patients during clinical follow-up and could be thoroughly registered in the RDR if needed [37].

### Checklist

The developed checklist, presented in Table 1, describes the recommended data elements to include in a disease-specific RDR intended to inform clinical trial design and enhance drug approval. It shows what type of data are needed, how often they have to be collected, the function of the data element, and some examples. Table 2 shows the comparison of the checklist with the two example registries. The ECFS registry is a well-established European oriented (28 countries) registry on cystic fibrosis. This disease mostly affects the lungs, and long-term issues include difficulty breathing and coughing up mucus as a result of frequent lung infections. The average life expectancy is between 42 and 50 years in the developed world [38, 39]. The main outcome collected is FEV1. In the evaluation of this RDR, it was shown that all areas in the checklist were included in the ECFS registry, except for the area on life impact (including outcomes such as health-related Quality of Life and daily functioning). The European DIPG registry collects information on diffuse intrinsic pontine glioma, an aggressive brain tumour of childhood located in the pons (middle of the brain stem). It affects the neurons within the spinal cord, as well as important structures involved in eye movements and in face and throat muscle control and sensation. The 5 years survival rate is less than 1% [40]. The DIPG registry predominantly collects outcome information on mortality. In this registry the area of life impact was not included either.

**Table 1:** Checklist: what type of information should be included in a RDR to inform clinical trial design

Type of information, proposed by experts (see references for more information)	Area(s)	Once (only re-entered when changed) or repeated?	Why needed	Applicability in drug approval process	Examples
Basic characterization [12]	Patient characteristics	Once, baseline	For (coded) identification of individual patients	Specific id per person is needed for all analyses	Patient id, date of birth
	Demographic characteristics	Once, baseline	To classify patient, information for patient retrieval, for trial feasibility assessment and/or to be aware of other factors possibly associated with outcome and to be able to adjust for it	For general ascertainment, historical controls, and post-marketing phase	Gender, age, ethnicity, country
Disease aspects [12]	Diagnosis	Once, baseline	To classify genotype and/or phenotype, for trial feasibility assessment and/or to be aware of other factors possibly associated with outcome and to be able to adjust for it	For general ascertainment, historical controls, and post-marketing phase	Date of first symptoms/diagnosis, genetic test results, type/staging of disease
	Co-morbidities	Once, baseline or when it is diagnosed, and if severity is worsening	For trial feasibility assessment and/or to be aware of other diagnoses possibly associated with disease and/or treatment effect,	For general ascertainment	Concurrent diagnoses, e.g. renal, cardiovascular or psychiatric problems
	Treatment	Repeated	For trial feasibility assessment and/or to be aware of other factors possibly associated with outcome and to be able to adjust for it	For general ascertainment, historical controls, post-marketing phase	Off-label treatment, surgery, dosage
Outcome variables (either for efficacy or safety) [14, 54]	Mortality	Once, when it occurs	To assess change in disease course over time (relevance depends on disease)/ To collect information on (un)expected, possible side-effects of treatment	For (Bayesian) sample size calculation, historical controls, and post-marketing phase	Survival time, age of death
	Life impact	repeated	To assess change in disease course over time at the personal level/ To collect information on (un)expected, possible side-effects of treatment	For (Bayesian) sample size calculation, historical controls, and post-marketing phase	Symptom status, functional status, general health perceptions, quality of life, cognitive functioning, hospitalization
	Pathophysiological manifestations	repeated	To assess change in disease course over time and phenotype /To collect information on (un)expected, possible side-effects of treatment	For (Bayesian) sample size calculation, historical controls, and post-marketing phase	Organ function, biomarkers, allergic reaction

**Table 2:** Comparison of the checklist with two example registries

Checklist elements	Area(s)	ECFS registry	Examples	DIPG registry	Examples
Basic characterization	Patient characteristics	+	Patient code	+	Patient ID
	Demographic characteristics	+	Gender, date of birth	+	Gender, ethnicity
Disease aspects	Diagnosis	+	First, second mutation	+	Cranial nerve palsy, eye movements, pathologic diagnosis
	Co-morbidities	+	Cause of death	+	
	Treatment	+	Antibiotics, pancreatic enzymes	+	Surgery, radiotherapy
Outcome variables (either for efficacy or safety)	Mortality	+	Survival	+	Survival
	Life impact	–		–	
	Pathophysiological manifestations	+	FEV1	+	Cranial nerve palsy, eye movements

## Discussion

Our research question focused on the possible applications of disease-specific RDRs for clinical trial design and regulatory approval and on the minimum information that should be recorded to maximize their potential for these purposes.

The results show that a RDR can be very helpful to improve the efficiency and quality of trial design in several ways. It can inform the sample size calculation, or, when prior information on the endpoints is available, even reduce the number of patients included. Besides informing a sample size calculation, the data of the RDR could, in certain circumstances, be used as an external control when placebo or active comparator groups are e.g. not ethically acceptable, and in larger RDRs, when of high quality, for registry-based RCTs. Also, in the post marketing phase a disease-specific RDR can be of value to supplement, confirm or (theoretically) refute data supporting the initial marketing authorization.

Designing a registry with a future clinical trial in mind can considerably reduce the time needed for the clinical development phase of a long awaited drug. One of the first steps in making a disease-specific RDR applicable to inform a drug trial for market authorization is to think of the research question and endpoints of that future trial. It is pivotal to consider what could be appropriate outcome measures in a very early stage, even when a trial is still far away. When it is still unclear what would be the best outcome measures, data from the RDR can help defining the most relevant ones. In addition to the goal(s) and desired outcome, factors need to be included that might influence the outcome but are not necessarily the focus of the study. When these potential confounders are also collected, their effect can be assessed and taken into account in the design of the study (i.e. used as stratification factors), but they could also be useful for matching historical cohorts [11]. The developed checklist provides a tool to check whether all types of important variables have been included. However, although the checklist has an expert and literature base, further validation of this tool by its actual use in designing rare disease trials is advised.

In the evaluation of two existing RDRs it was shown that data elements related to life impact were missing. Variables, with immediate relevance to patients, such as health related Quality of Life and variables concerning daily functioning are particularly important for patients and could improve RDR design and usefulness in a clinical trial. In Duchenne muscular dystrophy for example, patient advocacy groups identified the need to develop a scale to measure motor function of the upper limb to be able to also include non-ambulant children in the target group for new registration studies. The involvement of patients and families helped to select items reflecting clinically meaningful activities of daily living [41]. Therefore, it is advised to ascertain patient involvement in the development of a RDR so that patient-relevant outcome measures, generally considered very important by the regulators, are incorporated in the RDR. Besides, a RDR may represent opportunities for empowered patient organizations, who may initiate or sponsor registries as means to improve knowledge of certain rare diseases and to ease the conduct of clinical trials in certain conditions.

The use of validated measurement instruments and standardized measurement and data collection are other key aspects in RDR development, which prevent comparability issues later on when a trial is being set up. Several initiatives have been launched for standardization of outcome measurement [11]. One of these is the COMET initiative [42]. The COMET Initiative brings together researchers, clinicians and patients interested in the development and application of agreed standardized sets of outcomes, known as a ‘core outcome set.’ These sets represent the minimum that should be measured and reported in all clinical trials, audits of practice or other forms of research for a specific condition. This allows the results of trials and other studies to be compared and combined as appropriate. Besides the core outcome set, researchers are free to explore other outcomes as well. The International Consortium for Health Outcomes Measurement (ICHOM) is also aimed at harmonization of outcomes and data [43], in particular standardization of important outcomes of clinical care for patients on a global level. Although the application of these initiatives (predominantly in non-rare diseases) may be challenging for rare diseases, their methods could very well serve as a basis. Furthermore, new opportunities may lie in the rise of the European Reference Networks (ERNs), networks of centres of expertise and healthcare providers with a clear governance structure for knowledge sharing and care coordination across borders [44]. The ERNs, focusing on specific (clusters of) disease(s), can unite relevant stakeholders, including patient representatives and could initiate standardization to improve comparability and data linkage and sharing of RDR data.

With regard to the instruments used to measure the relevant outcomes, the measurement properties, such as reliability, validity, and responsiveness need to be assessed and found adequate before they can be used in clinical trials. Important decisions are based on the results obtained with these instruments; therefore one needs to be confident that these results are reliable and valid. The US Food and Drug Administration (FDA) and EMA require that measurement instruments are well validated for their purpose [45, 46]. Especially for outcome measures that could have a subjective nature, such as patient-reported outcomes, it is important to evaluate the reliability and validity of these instruments. If no useful measurement instruments exist to measure the effect of interventions in a particular disease, the development and validation of measurement instruments should start as early as possible, since this process takes considerable time, especially in rare diseases.

The importance of longitudinal data collection for the applicability of RDR in clinical trials has been stipulated. The key strength of collecting an outcome measure at multiple follow-up times is the possibility to measure individual change in outcome, which enables comparison between different patient groups. Although we strongly recommend collecting data in a longitudinal manner, this type of data collection is costly and requires a high level of organization, such as in logistics and personnel. To financially sustain a basic RDR is already challenging, so it needs a creative approach to keep such a registry running and maintain a high quality data collection [47]. For example, agreeing on standardized approaches to collect medical information into electronic medical records during routine clinical practice may be a strategy that can be explored in order to improve the feasibility and efficiency of a RDR. For the statistical analysis of longitudinal data specific methods are required that properly adjust for the intra-subject correlation between the measurements [48, 49].

Although the possible benefit of a RDR in the post-marketing phase has been described, some caution has to be put in place as well. Especially regarding efficacy assessment after drug approval in populations not evaluated in the trial. Some may argue that systematic compassionate use after market approval may weaken the robustness of information supporting regulatory and clinical decision making in these neglected populations, as it may preclude the conduction of randomized trials able to conclude efficacy. Meanwhile, in certain small subgroups the comparison with RDR data for enlargement of the market authorization might be one of the few possibilities left. Therefore, careful deliberation on the pros and cons of different scenarios is desirable.

### Future directions

The option of reducing the required sample size of a RCT in (very) rare diseases by using a RDR as historical control group is under debate. In very small populations with life threatening or seriously disabling diseases it may be the only ethically acceptable approach and it is worthwhile to further investigate its options.

At this moment, alternatives for a RCT such as historical controls are only taken into consideration in specific circumstances, and acceptability by regulators is determined on a case by case basis. Aspects like limited life-expectancy, limited or no availability of current treatments, and expected magnitude of the treatment effect could be possible reasons to consider the possibility of using information from a RDR as comparator [50].

The regulatory agencies often ask for longitudinal data collection after (conditional) drug approval to assess safety on the long term [51]. Several commercial companies comply with this request by setting up specific product or drug registries, in which only patients using the drug are included. In our opinion this post-marketing safety assessment should be conducted by means of (already existing) disease-specific RDRs. Besides the fact that most of the time, safety parameters are already included (saving time and money), disease-specific registries, rather than product-specific registries, may be useful to compare effectiveness in a clinical setting across types of patients and treatments, and could protect the evaluation process from commercial influences [11, 52]. Furthermore, the information on a rare disease could then be collected in one place, instead of being divided among several commercial companies who might be unwilling to share ‘their’ data [53]. Collaboration between stakeholders, such as health authorities, (several) pharmaceutical companies, academic researchers and rare disease patient organizations to develop feasible strategies for this matter should be the first priority aiming at a considerable improvement in achieving sustainable longitudinal RDRs, which may ultimately enhance the speed of getting effective orphan drugs to those who need them.

## Conclusions

A RDR can be very helpful in trial design by informing the sample size calculation, it can increase efficiency by being a data collection tool in clinical trials, may provide a historical control group in instances when placebo or active comparators are e.g. not ethically acceptable, and it can be informative in the post marketing phase.

To enable the applicability and optimal use of a RDR longitudinal data collection is indispensable, and specific data collection, prepared for repeated measurement, is needed. The developed checklist can help to define the appropriate variables to include.

Disease-specific RDRs are preferred over product-specific registries. In a disease-specific RDR all consenting patients with the disease are included, and not only the patients who receive a certain treatment.

Valid measurement instruments should be used, and measurements, data collection and data management should make use of global data standards to optimise comparability with clinical trial data.

### Agenda for researchers, regulators and funders with regard to RDRs

Prior information used as observations in the new trial, or combining RDR data and trial data could be a possibility to reduce the sample size of a rare disease trial under certain conditions. More research is needed to define the circumstances in which this approach could be used and what are the content requirements.

There are some examples where RDR data, either collected retrospectively or prospectively, were used to replace the use of placebo or active comparators. However, for many patients and patient organizations, as well as for many scientists, it is not clear in what circumstances this might or might not be acceptable. A description of situations in which the use of historical data in trial design might be acceptable would be helpful for future RDR builders to foresee the (im)possibilities of the RDR use and what should be taken into account for that.

To maximize efficiency, post-marketing safety assessment should be conducted by means of (already existing) disease-specific registries instead of product-specific registries, not only to allow for comparisons and protect the evaluation process from commercial influences, but also to enhance possibilities for gathering information on the use of the products in special circumstances (such as extended licensing) or even for collecting clinical trial data. Health authorities, pharmaceutical companies, researchers and patients should make this a common accomplishment.

It is recommended to conduct an international consensus procedure on the content, inclusion criteria, governance, traceability of, and access to disease-specific RDRs needed for post-marketing surveillance.

## Abbreviations

Asterix: Advances in Small Trials dEsign for Regulatory Innovation and eXcellence
CDE: Common data elements
CHMP: Committee for medicinal products for human use
DIPG: Diffuse intrinsic pontine glioma
ECFS: European Cystic Fibrosis Society
EPAR: European Public Assessment Report
ERN: European Reference Network
FDA: US Food and Drug Administration
FEV: Forced expiratory volume
ICHOM: International Consortium for Health Outcomes Measurement
RCT: Randomized controlled trial
RDR: Rare disease registry
VOD: Veno-occlusive disease

## References

[1] Forrest CB, Bartek RJ, Rubinstein Y, Groft SC (2011). The case for a global rare-diseases registry. Lancet.

[2] van der Lee JH, Tanck MW, Wesseling J, Offringa M (2009). Pitfalls in the design and analysis of paediatric clinical trials: a case of a ‘failed’ multi-centre study, and potential solutions. Acta Paediatr.

[3] Nikolakopoulos S, Roes KC, van der Lee JH, van der Tweel I (2014). Sample size calculations in pediatric clinical trials conducted in an ICU: a systematic review. Trials.

[4] Joppi R, Bertele V, Garattini S (2013). Orphan drugs, orphan diseases. The first decade of orphan drug legislation in the EU. Eur J Clin Pharmacol.

[5] European Organisation for Rare Diseases (EURORDIS) (2005). Rare diseases: understanding this public health priority.

[6] Orphanet report series: rare disease registries in Europe - May 2017. http://www.orpha.net/orphacom/cahiers/docs/GB/Registries.pdf. Accessed 30 Apr 2018.

[7] Bateman RJ, Benzinger TL, Berry S, Clifford DB, Duggan C, Fagan AM, Fanning K, Farlow MR, Hassenstab J, McDade EM (2017). The DIAN-TU next generation Alzheimer’s prevention trial: adaptive design and disease progression model. Alzheimers Dement.

[8] Santoro M, Coi A, Lipucci Di Paola M, Bianucci AM, Gainotti S, Mollo E, Taruscio D, Vittozzi L, Bianchi F (2015). Rare disease registries classification and characterization: a data mining approach. Public Health Genomics.

[9] Dolk H, Loane M, Teljeur C, Densem J, Greenlees R, McCullough N, Morris J, Nelen V, Bianchi F, Kelly A (2015). Detection and investigation of temporal clusters of congenital anomaly in Europe: seven years of experience of the EUROCAT surveillance system. Eur J Epidemiol.

[10] Tyczynski JE, Démaret E, Parkin DM (2003). Standards and guidelines for cancer registration in Europe.

[11] Gliklich RE, Dreyer N, Leavy M (2014). Registries for evaluating patient outcomes: a user’s guide.

[12] Taruscio D, Mollo E, Gainotti S, Posada de la Paz M, Bianchi F, Vittozzi L (2014). The EPIRARE proposal of a set of indicators and common data elements for the European platform for rare disease registration. Arch Public Health.

[13] Hilgers RD, Roes K, Stallard N, IdeAl A, In Spg (2016). directions for new developments on statistical design and analysis of small population group trials. Orphanet J Rare Dis.

[14] Boers M, Kirwan JR, Wells G, Beaton D, Gossec L, d'Agostino MA, Conaghan PG, Bingham CO, Brooks P, Landewe R (2014). Developing core outcome measurement sets for clinical trials: OMERACT filter 2.0. J Clin Epidemiol.

[15] Tabrizi SJ, Reilmann R, Roos RA, Durr A, Leavitt B, Owen G, Jones R, Johnson H, Craufurd D, Hicks SL (2012). Potential endpoints for clinical trials in premanifest and early Huntington's disease in the TRACK-HD study: analysis of 24 month observational data. Lancet Neurol.

[16] Lauer MS, D’Agostino RB (2013). The randomized registry trial--the next disruptive technology in clinical research?. N Engl J Med.

[17] Relton C, O’Cathain A, Nicholl J (2012). A pilot ‘cohort multiple randomised controlled trial’ of treatment by a homeopath for women with menopausal hot flushes. Contemp Clin Trials.

[18] World Medical Association: Declaration of Helsinki. https://www.wma.net/policies-post/wma-declaration-of-helsinki-ethical-principles-for-medical-research-involving-human-subjects/. Accessed 13 Nov 2017.

[19] International council for harmonisation of technical requirements for pharmaceuticals for human use (2016). ICH E6 Good Clinical Practice (GCP) Guideline.

[20] Kalkman S, van Thiel G, Zuidgeest MGP, Goetz I, Pfeiffer BM, Grobbee DE, van Delden JJM, Work Package 3 of the IMIGC (2017). Series: pragmatic trials and real world evidence: paper 4. Informed consent. J Clin Epidemiol.

[21] Li G, Sajobi TT, Menon BK, Korngut L, Lowerison M, James M, Wilton SB, Williamson T, Gill S, Drogos LL (2016). Registry-based randomized controlled trials- what are the advantages, challenges, and areas for future research?. J Clin Epidemiol.

[22] European Medicines Agency (2007). Guideline on clinical trials in small populations.

[23] Moher D, Hopewell S, Schulz KF, Montori V, Gotzsche PC, Devereaux PJ, Elbourne D, Egger M, Altman DG, Consolidated Standards of Reporting Trials G (2010). CONSORT 2010 explanation and elaboration: updated guidelines for reporting parallel group randomised trials. J Clin Epidemiol.

[24] van der Ploeg AT, Clemens PR, Corzo D, Escolar DM, Florence J, Groeneveld GJ, Herson S, Kishnani PS, Laforet P, Lake SL (2010). A randomized study of alglucosidase alfa in late-onset Pompe’s disease. N Engl J Med.

[25] European Medicines Agency: Myozyme, European Public Assessment Report. http://www.ema.europa.eu/ema/index.jsp%3Fcurl%3Dpages/medicines/human/medicines/000636/human_med_000917.jsp%26murl%3Dmenus/medicines/medicines.jsp. Accessed 10 June 2016.

[26] Nicolino M, Byrne B, Wraith JE, Leslie N, Mandel H, Freyer DR, Arnold GL, Pivnick EK, Ottinger CJ, Robinson PH (2009). Clinical outcomes after long-term treatment with alglucosidase alfa in infants and children with advanced Pompe disease. Genet Med.

[27] Kishnani PS, Corzo D, Nicolino M, Byrne B, Mandel H, Hwu WL, Leslie N, Levine J, Spencer C, McDonald M (2007). Recombinant human acid [alpha]-glucosidase: major clinical benefits in infantile-onset Pompe disease. Neurology.

[28] Byrne BJ, Kishnani PS, Case LE, Merlini L, Muller-Felber W, Prasad S, van der Ploeg A (2011). Pompe disease: design, methodology, and early findings from the Pompe registry. Mol Genet Metab.

[29] Kishnani PS, Hwu WL, Mandel H, Nicolino M, Yong F, Corzo D, Infantile-Onset Pompe Disease Natural History Study G (2006). A retrospective, multinational, multicenter study on the natural history of infantile-onset Pompe disease. J Pediatr.

[30] European Medicines Agency: Myozyme, Scientific discussion. http://www.ema.europa.eu/docs/en_GB/document_library/EPAR_-_Scientific_Discussion/human/000636/WC500032128.pdf. Accessed 10 June 2016.

[31] European Medicines Agency: Defitelio, European Public Assessment Report. http://www.ema.europa.eu/ema/index.jsp?curl=pages/medicines/human/medicines/002393/human_med_001646.jsp&mid=WC0b01ac058001d124. Accessed 10 June 2016.

[32] European Medicines Agency: Elaprase, European Public Assessment Report. http://www.ema.europa.eu/docs/en_GB/document_library/EPAR_-_Summary_for_the_public/human/000700/WC500023003.pdf. Accessed 10 Sept 2017.

[33] Giugliani R, Hwu WL, Tylki-Szymanska A, Whiteman DA, Pano A (2014). A multicenter, open-label study evaluating safety and clinical outcomes in children (1.4-7.5 years) with hunter syndrome receiving idursulfase enzyme replacement therapy. Genet Med.

[34] Muenzer J, Jones SA, Tylki-Szymanska A, Harmatz P, Mendelsohn NJ, Guffon N, Giugliani R, Burton BK, Scarpa M, Beck M (2017). Ten years of the Hunter Outcome Survey (HOS): insights, achievements, and lessons learned from a global patient registry. Orphanet J Rare Dis.

[35] Weinreb NJ, Kaplan P (2015). The history and accomplishments of the ICGG Gaucher registry. Am J Hematol.

[36] Weinreb NJ, Charrow J, Andersson HC, Kaplan P, Kolodny EH, Mistry P, Pastores G, Rosenbloom BE, Scott CR, Wappner RS, Zimran A (2002). Effectiveness of enzyme replacement therapy in 1028 patients with type 1 Gaucher disease after 2 to 5 years of treatment: a report from the Gaucher registry. Am J Med.

[37] Kerem E, Viviani L, Zolin A, MacNeill S, Hatziagorou E, Ellemunter H, Drevinek P, Gulmans V, Krivec U, Olesen H, Group EPRS (2014). Factors associated with FEV1 decline in cystic fibrosis: analysis of the ECFS patient registry. Eur Respir J.

[38] Ong T, Ramsey BW (2015). Update in cystic fibrosis 2014. Am J Respir Crit Care Med.

[39] O’Sullivan BP, Freedman SD (2009). Cystic fibrosis. Lancet.

[40] Donaldson SS, Laningham F, Fisher PG (2006). Advances toward an understanding of brainstem gliomas. J Clin Oncol.

[41] Mayhew A, Mazzone ES, Eagle M, Duong T, Ash M, Decostre V, Vandenhauwe M, Klingels K, Florence J, Main M (2013). Development of the performance of the upper limb module for Duchenne muscular dystrophy. Dev Med Child Neurol.

[42] Williamson P, Clarke M. The COMET (Core Outcome Measures in Effectiveness Trials) initiative: its role in improving Cochrane reviews. Cochrane Database Syst Rev. 2012;(4):ED000041. 10.1002/14651858.ED000041. 10.1002/14651858.ED000041 PMC10846459 22592744

[43] International Consortium for Health Outcomes Measurement: ICHOM initiative. http://www.ichom.org/. Accessed 12 Sept 2016.

[44] European Commision: European Reference Networks. https://ec.europa.eu/health/ern/policy_en. Accessed 10 Sept 2017.

[45] U.S. Department of Health and Human Services Food and Drug Administration: Clinical Outcome Assessment Qualification Program https://www.fda.gov/drugs/developmentapprovalprocess/drugdevelopmenttoolsqualificationprogram/ucm284077.htm. Accessed 12 June 2017.

[46] European Medicines Agency (2005). Reflection paper on the regulatory guidance for the use of health related quality of life (HRQL)measures in the evaluation of medicinal products.

[47] Viviani L, Zolin A, Mehta A, Olesen HV (2014). The European cystic fibrosis society patient registry: valuable lessons learned on how to sustain a disease registry. Orphanet J Rare Dis.

[48] Hardin J, Hilbe J (2003). Generalized estimating equations.

[49] Zeger SL, Liang KY (1986). Longitudinal data analysis for discrete and continuous outcomes. Biometrics.

[50] European Medicines Agency (2001). Note for guidance on choice of control group in clinical trials.

[51] Bouvy JC, Blake K, Slattery J, De Bruin ML, Arlett P, Kurz X (2017). Registries in European post-marketing surveillance: a retrospective analysis of centrally approved products, 2005-2013. Pharmacoepidemiol Drug Saf.

[52] Schmitt-Egenolf M (2006). Psoriasis therapy in real life: the need for registries. Dermatology.

[53] Hollak CE, Aerts JM, Ayme S, Manuel J (2011). Limitations of drug registries to evaluate orphan medicinal products for the treatment of lysosomal storage disorders. Orphanet J Rare Dis.

[54] Wilson IB, Cleary PD (1995). Linking clinical variables with health-related quality of life. A conceptual model of patient outcomes. JAMA.

